# Stentless Root Replacement *versus* Tissue Valves in Infective Endocarditis - A Propensity-Score Matched Study

**DOI:** 10.21470/1678-9741-2020-0267

**Published:** 2020

**Authors:** Jerry Easo, Marcin Szczechowicz, Philipp Hölzl, Adrian Meyer, Konstantin Zhigalov, Rizwan Malik, Rohit Philip Thomas, Alexander Weymann, Otto E. Dapunt

**Affiliations:** 1 Department of Cardiac Surgery, University Clinic Oldenburg, European Medical School Oldenburg-Groningen, Oldenburg, Germany.; 2 Division of Cardiac Surgery, Medical University of Graz, Graz, Austria.; 3 Department of Thoracic and Cardiovascular Surgery, Essen University Hospital, Essen Germany.; 4 Department of Diagnostic and Interventional Radiology, Philipps-University Marburg, Marburg, Germany.

**Keywords:** Reoperation, Hospital Mortality, Follow-Up Studies, Odds Ratio, Endocarditis, Cohort Studies, Aorta, Bacteremia, Coronary Disease

## Abstract

**Introduction:**

People with aortic/prosthetic valve endocarditis are a high-risk cohort of patients who present a challenge for all medically involved disciplines and who can be treated by various surgical techniques.

**Methods:**

We analyzed the results of treatment of root endocarditis with Medtronic Freestyle® in full-root technique over 19 years (1999-2018) and compared them against treatment with other tissue valves. Comparison was made with propensity score matching, using the nearest neighbor method. Various tests were performed as suited for adequate analyses.

**Results:**

Fifty-four patients in the Medtronic Freestyle group (FS group) were matched against 54 complex root endocarditis patients treated with other tissue valves (Tissue group). Hospital mortality was 9/54 (16.7%) in the FS group *vs*. 14/54 (25.6%) in the Tissue group (*P*=0.24). Cox regression performed for early results demonstrated coronary heart disease (*P*=0.004, odds ratio 2.3), among others, influencing early mortality. Recurrent infection was low (1.8% for FS and Tissue patients) and freedom from reoperation was 97.2% at a total of 367 patient-years of follow-up (median of 2.7 years).

**Conclusion:**

The stentless xenograft is a viable alternative for treatment of valve/root/prosthetic endocarditis, demonstrating a low rate of reinfection. The design of the bioroot allows for complex reconstructive procedures at the outflow tract and the annular level with at an acceptable operative risk. Endocarditis patients can be treated excluding infective tissue from the bloodstream, possibly with benefits, concerning bacteremia and recurrent infection. Furthermore, the use of the stentless bioroot offers varying treatment options in case of future valve degeneration.

**Table:** 

Abbreviations, acronyms & symbols	
AVR	= Aortic valve replacement
CABG	= Coronary artery bypass grafting
CHD	= Congestive heart disease
CI	= Confidence interval
CPB	= Cardiopulmonary bypass
ECMO	= Extracorporeal membrane oxygenator
FS	= Freestyle
HR	= Hazard ratios
IABP	= Intra-aortic balloon pump
ICU	= Intensive care unit
LOS	= Low output syndrome
MV	= Mitral valve
NVE	= Native valve endocarditis
OR	= Odds ratios
PVE	= Prosthetic valve endocarditis

## INTRODUCTION

Treatment of aortic root endocarditis remains a daunting and challenging procedure, often complicated by periannular abscess formation and dehiscence of the mitral-aortic continuity. Patients with infective endocarditis represent a high-risk cohort with significant mortality^[1,2]^, and early diagnosis/radical surgical treatment in the acute phase of endocarditis remains the gold standard treatment. Aggressive debridement of infected tissue is mandatory, often leading to severe defects of the outflow tract requiring complex reconstructive procedures^[3,4]^. The debate about prosthesis choice in this setting has been ongoing and is dependent on several factors influencing long-term performance. Homografts are recommended by the international guidelines^[2]^, stentless valves have proven their efficacy in smaller case series with excellent long-term results^[5,6]^, and conventional stented valves are a further option in the armamentarium of surgical procedures for valve endocarditis^[7,8]^.

We accessed data from our Medtronic Freestyle® valve patients, investigating the cohort of patients with infective endocarditis, either as native valve endocarditis (NVE) or prosthetic valve endocarditis (PVE), treated with the stentless xenograft, analyzing early and long-term results. We performed a propensity score matching analysis comparing results against a cohort of patients treated for complex aortic root and prosthetic valve infection with other tissue valves over the same time interval, identifying risk factors for in-hospital and long-term mortality. Furthermore, we performed a comparative analysis for the occurrence of postoperative complications and recurrence of graft infection or necessary reoperations.

## METHODS

From the Oldenburg Freestyle database, patients with infective endocarditis were identified and included in the FS group. These patients suffered from NVE or PVE. Definition of endocarditis was according to positive blood culture, echocardiographic signs, vegetation, and/or root abscess according to the modified Duke criteria. Germ identification was performed, and relevant antibiotic treatment was initiated as soon as possible. Surgical treatment was applied in all patients in the acute phase of treatment and was performed by eight attending surgeons of the authors’ institution. Stentless bioroots were implanted in a full-root technique in all FS patients.

Patients with complex root endocarditis treated with other tissue valves were identified from our entire cohort of patients over the time span of 19 years (01.01.1999 to 15.08.2018). These included homografts, biological conduits other than Freestyle, and conventional stented xenografts. Stented tissue valves were used with abscess cavity closure by glutaraldehyde fixated pericardial closure or by inclusion via the proximal suturing of the xenograft.

**Fig.1:**
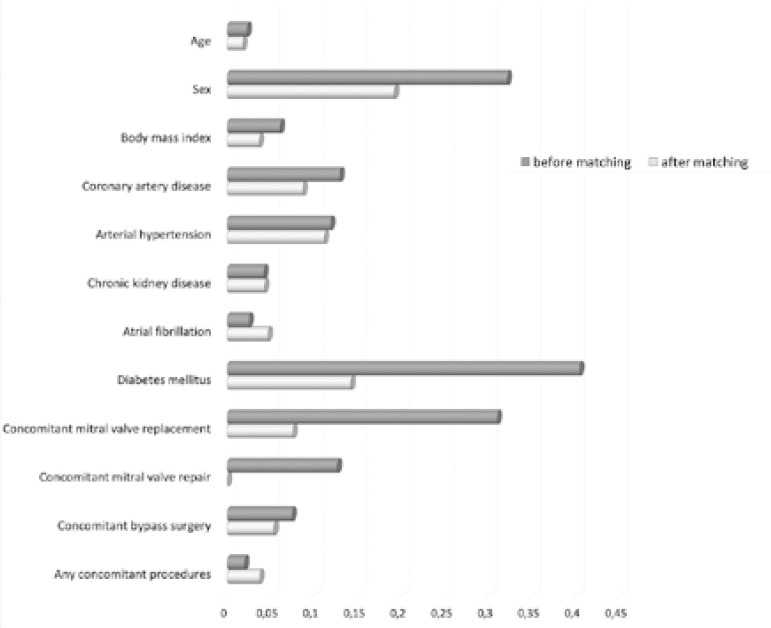
Standardized mean differences of matching variables comparing Freestyle and Tissue groups.

To perform a comprehensive comparative analysis, a propensity score matching was applied, using the nearest neighbor method to achieve small mean standardized differences and identifying a control group of 54 patients (Figure 1). The variables included paired matching of age, sex, body mass index, coronary artery disease, arterial hypertension, chronic kidney disease, atrial fibrillation, diabetes, concomitant mitral valve surgery, concomitant bypass surgery, and any other concomitant procedures.

Echocardiographic examination with vegetations on native or prosthetic valves, presence of intra- or periannular abscess, valve regurgitation, and atrioventricular dehiscence/fistulae were documented. Analysis of operative variables was performed with investigation of relevant cardiopulmonary bypass times, operative mortality, sepsis, and renal insufficiency as well as new onset of neurological deficits, among others. Primary endpoints were death and valve-related complications, including reinfection or reoperation of the implanted valve. New onset of thromboembolic complications or bleeding as well as structural valve deterioration were documented.

Clinical data was collected with reporting of adverse events according to the recommended guidelines of the Society of Thoracic Surgery and the American Association of Thoracic Surgery^[9]^. Hospital mortality was defined as mortality within the initial 30-day postoperative period or within the hospital stay when exceeding the first 30 days. Approval of the Ethics Committee of the University of Oldenburg was obtained (Ethics Approval Number 2017-040) with waiving of signed consent form due to the retrospective data analysis. The clinical data included in the postoperative follow-up were obtained by telephone questionnaire. Composite of death, reoperation, reinfection, and stroke was created to analyze major adverse events. Follow-up started upon date of discharge and was performed until death or the chosen cut-off period (15.08.2018). A follow-up of 100% was achieved.

The continuous variables were presented as means ± standard deviations if normally distributed. In case of non-normal distribution, the data was presented as medians with quartiles. Distributions were checked with Shapiro-Wilk and Kolmogorov-Smirnov tests. Independent sample *t*-tests were performed for normally distributed mean comparison and non-parametric tests in cases of non-normal distribution. Categorical variables were presented as absolute values and percentages with use of the chi-square test for comparison. Univariate regression analysis with presentation as odds ratios (OR) with 95% confidence intervals (CI) were implemented for assessing the risk-factors for early mortality. Risk factor analysis for late mortality was performed with use of the proportional hazard model with presentation as hazard ratios (HR) with 95% CI. Survival analysis was performed using the Kaplan-Meier method and survival comparison was by implementation of the Log-Rank and Breslow tests.

We performed the statistical analysis using IBM Corp. Released 2017, IBM SPSS Statistics for Windows, Version 25, Armonk, NY: IBM Corp. and R software v.3.4.3 (R Foundation for Statistical Computing, Vienna, Austria).

## RESULTS

The entire cohort of Medtronic Freestyle patients undergoing surgery at our institution consisted of 971 patients over 19 years. From this collective, a subgroup of 54 acute infective root endocarditis patients (the FS group) were identified. The entire unmatched group of tissue valve patients treated for endocarditis consisted of 331 patients; 171 could be identified with a complex root pathology including periannular abscess formation, intracardiac fistula, and aortomitral discontinuity in NVE or PVE. Fifty-four patients (defined as the Tissue group) were identified from this cohort using retrospective propensity matching.

Table 1 demonstrates the preoperative demographic data, similar in all variables. Tissue valve patients were of similar age to FS patients (mean of 65.8±12.6 *vs*. 62.8±13.3 years, respectively; *P*=0.20). Table 2 demonstrates further endocarditis characteristics, with staphylococcal strains as the main contributor and abscess formation in over 40% of the patients treated with the xenograft bioroot. Four patients (7.4%) in both groups had intracardiac fistula formation requiring closure, two redo FS patients and two redo Tissue patients. Four patients in the FS group and four patients in the Tissue valve group required patchplasty for treatment of the tissue defects created by the debridement necessary for definitive treatment of the endocarditis.

**Table 1:** Preoperative patients' characteristics.

Characteristics	Unmatched (n=331)	Freestyle (n=54)	Tissue (n=54)	*P*-value
Demographic data				
Number of patients	331	54	54	1.0
Age (years)	66.39±12.5	62.8±13.3	65.8±12.6	0.20
Female	85 (25.7%)	8 (14.8 %)	12 (22.2%)	0.32
Body surface area (m^2^)	1.96±0.23	1.97±0.19	1.96±0.17	0.80
Aneurysm of the aorta ascendens	11 (3.3%)	2 (3.7%)	4 (7.4%)	0.40
Aortic dissection	5 (1.2%)	1 (1.8%)	1 (1.8%)	1.0
Coronary artery disease	89 (26.8%)	12 (22.2%)	14 (25.9%)	0.65
Arterial hypertension	137 (41.3%)	25 (46.2%)	28 (51.8%)	0.56
Diabetes	54 (16.3%)	3 (5.5%)	5 (9.2%)	0.46
Chronic kidney disease	91 (27.4%)	14 (25.9%)	13 (24.1%)	0.82
History of cardiac surgery	133 (40.1%)	34 (62.9%)	24 (44.4%)	0.25
Previous Freestyle	22 (5.1%)	6 (11.1%)	4 (7.4%)	0.51
Previous aortic valve replacement	127 (38.3%)	34 (62.9%)	24 (44.1%)	0.08
Previous mitral valve surgery	1 (0.5%)	0	0	1.0
Previous bypass surgery	19 (5.7%)	3 (5.5%)	5 (9.2%)	0.46
Other	2 (0.6%)	0	0	1.0

**Table 2:** Characteristics of endocarditis.

Characteristics	Freestyle (n=54)	Tissue (n=54)	*P*-value
Presence of abscess	24 (44.4%)	15 (27.7%)	0.07
Presence of intracardiac fistula	4 (7.4%)	4 (7.4%)	1.0
Aortomitral dehiscence	15 (27.7%)	10 (18.5%)	0.25
*Staphylococcus*	23 (42.5%)	17 (31.4%)	0.23
*Streptococcus*	3 (5.5%)	5 (9.2%)	0.46
*Enterococcus*	11 (20.3%)	12 (22.2%)	0.81
No germ detected	16 (29.6%)	16 (29.6%)	1.0
Other	2 (3.7%)	4 (7.4%)	0.07

Table 3 shows intraoperative data and adverse postoperative outcomes. Forty-nine Tissue patients were treated with stented xenografts and five patients with other biological conduits (three with Vascutek Root Elan^®^, one with BioIntegral^®^, and one with the Shelhigh NoReact^®^ conduit). There were no major differences in the intraoperative incidence of concomitant surgery; the replacement of the ascending aorta being more often in the FS group (*P*=0.008). The root replacement procedure was reflected by longer periods of cardiopulmonary bypass and respective cross-clamp time.

**Table 3:** Intraoperative variables and postoperative adverse outcomes.

Characteristics	Unmatched (n=331)	Freestyle (n=54)	Tissue (n=54)	*P*-value
Conduit	75 (22.6%)	54 (100%)	5 (9.3%)	0.0001
Homograft	11 (2.6%)	0	0	1.0
+ MV- surgery	80 (24.1%)	7 (12.9%)	9 (16.6%)	0.59
+ MV replacement	44 (14.4%)	3 (5.5%)	4 (7.4%)	0.7
+ MV reconstruction	12 (3.6%)	1 (1.8%)	1 (1.8%)	1.0
+ Ascending aorta	20 (6.0%)	11 (20.3%)	2 (3.7%)	0.008
+ Bypass	42 (12.6%)	8 (14.8%)	7 (12.9%)	0.78
+ Bailout bypass	8 (2.4%)	3 (5.5%)	1 (1.8%)	0.31
Operation time (min)	202.6±101.6	292.9±84.2	191.5±95.2	<0.001
CPB time (min)	124.7±75.6	191±66.9	143.2±70.1	<0.001
Cross-clamp time (min)	80.6±42.4	125.7±33.3	80.8±32.3	<0.001
ECMO	12 (3.6%)	3 (5.5%)	4 (7.4%)	0.69
IABP	20 (6.1%)	7 (12.9%)	5 (9.3%)	0.54
Acute kidney injury	60 (18.1%)	10 (18.5%)	12 (22.2%)	0.63
Atrial fibrillation	61 (18.4%)	12 (22.2%)	14 (25.9%)	0.65
Rethoracotomy	29 (8.7%)	7 (12.9%)	4 (7.4%)	0.34
Stroke	5 (1.5%)	1 (1.8%)	1 (1.8%)	1.0
LOS	17 (5.1%)	3 (5.5%)	1 (1.8%)	0.31
Postop pacemaker	31 (9.4%)	7 (22.2%)	3 (5.5%)	0.18
In-hospital mortality	62 (18.7%)	9 (16.7%)	14 (25.9%)	0.24

CPB=cardiopulmonary bypass; ECMO=extracorporeal membrane oxygenator; IABP=intra-aortic balloon pump; ICU=intensive care unit; LOS=low output syndrome; MV=mitral valve

In-hospital mortality was numerically lower for FS patients in comparison to Tissue patients, however statistically failing to reach any significance. An analysis of the 14 deceased Tissue patients (25.9%) demonstrated devastating preoperative conditions of two patients in cardiogenic and septic shock and seven patients with reoperative surgery; three patients had double valve replacement with involvement of left and right sided valves and three patients with root surgery performed had concomitant surgery with bypass grafting. From the nine patients in the FS group (16.7%), a similar preoperative high-risk profile could be seen with eight reoperative procedures, two patients with double valve endocarditis, and one patient with left ventricular-right atrial fistula formation.

Follow-up was performed as described above. The median follow-up period was 2.7 years, with a total of 367 patient-years. Logistic regression analysis using a univariate model was performed to determine risk factors for in-hospital mortality. Age (*P*=0.04, OR 1.05, 95% CI 1.00-1.109) and congestive heart disease (CHD) (*P*=0.004, OR 4.23, 95% CI 1.59-11.5) were preoperative variables with influence on in-hospital mortality. Postoperative onset of renal insufficiency (*P*=0.001, OR 14.9, 95% CI 4.93-45.41) influenced early mortality. History of previous cardiac surgery posed no significance in the analysis for risk factors (*P*=0.09). Likewise, history of Freestyle implantation showed no significant risk for in-hospital mortality (*P*=0.32).

In a similar fashion, Cox regression analysis was used to analyze risk factors contributing to long-term mortality (Table 4). Age (*P*=0.008, HR 1.05, 95% CI 1.011-1.079) and CHD showed significance (*P*=0.009, HR 2.26, 95% CI 1.24-4.5). The necessity for bypass surgery (*P*=0.001, HR 3.7, 95% CI 1.85-7.5) and postoperative renal impairment (*P*=0.001, HR 6.4, CI 95% 3.26-12.7) showed statistic relevance. Furthermore, annular dehiscence demonstrated a 3,5-fold risk for late and 8-fold risk for early mortality.

**Table 4:** Cox regression and logistic regression analysis for early and long-term results.

	In-hospital mortality				Long-term mortality			
			95% CI for OR				95% CI for HR	
Characteristics	*P*-value	Odds ratio	Lower	Upper	*P*-value	Hazard ratio	Lower	Upper
Freestyle					0.367	0.764	0.425	1,372
CHD	0.004	4,278	1,589	11,514	0.009	2,366	1,236	4,53
Operation time	0.000	1,009	1,004	1,014	0.000	1,006	1,004	1,009
CPB time	0.000	1,014	1,007	1,021	0.000	1,009	1,005	1,013
Cross-clamp time	0.026	1,013	1,002	1,024	0.065	1,007	1	1,014
CABG	0.002	5,943	1,872	18,865	0.000	3,715	1,845	7,478
Bailout CABG	0.854	1,242	0.123	12,537	0.180	2,237	0.689	7,261
Postoperative renal impairment	0.000	14,972	4,936	45,415	0.000	6,428	3,257	12,688
Postoperative tamponade	0.030	5,754	1,188	27,88	0.002	3,868	1,61	9,296
Drainage loss	0.003	1	1	1	0.003	1	1	1
Ventilation	0.002	1,005	1,002	1,008	0.000	1,003	1,002	1,004
Previous surgery	0.093	2,402	0.863	6,687	0.058	1,846	0.980	3,48
Previous FS	0.317	0.341	0.041	2,811	0.361	0.579	0.179	1,872
Previous AVR	0.132	2,13	0.796	5,702	0.150	1,565	0.850	2,882
Fistula	0.054	4,263	0.977	18,601	0.114	2,13	0.833	5,448
Dehiscence	0.000	7,908	2,835	22,06	0.000	3,488	1,824	6,671

AVR=aortic valve replacement; CABG=coronary artery bypass grafting; CHD=congestive heart disease; CI=confidence interval; CPB=cardiopulmonary bypass; FS=Freestyle

**Fig. 2:**
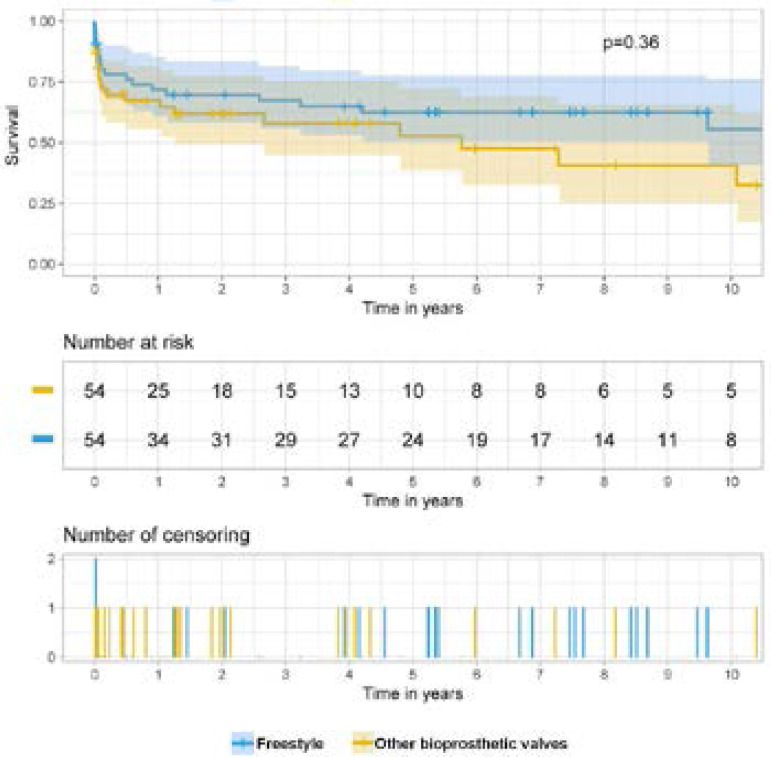
Kaplan-Meier survival curve with comparison between Freestyle and Tissue groups.

For survival analysis, the Kaplan-Meier method was used (Figure 2). Cumulative survival at one, three, five, and ten years in the FS group was 70%, 66%, 63%, and 49% *versus* 60%, 57%, 47% and 29%, respectively, in the non-FS group. Log Rank test (Mantel Cox) was insignificant (*P*=0.36), and Breslow test demonstrated likewise no significant difference in long-term survival (*P*=0.33).

**Fig. 3:**
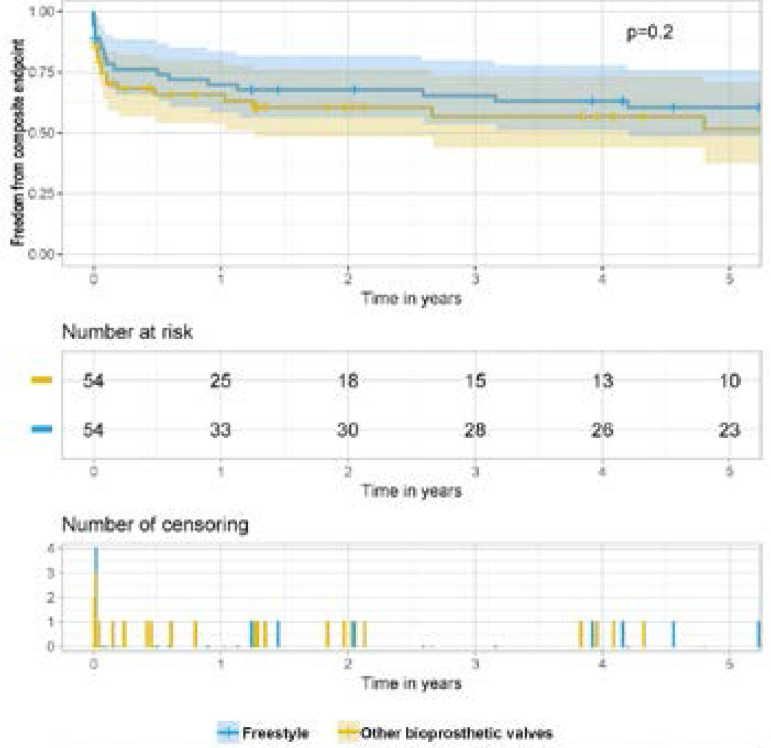
Kaplan-Meier curve of composite endpoint (death, stroke, reoperation, and valve infection) of Freestyle and Tissue groups

Follow-up demonstrated a recurrent infection in 1/54 patients in the FS group, treated with a second stentless bioroot (1.8%), and 1/54 in the Tissue group (1.8%), treated with full-root replacement. There was no structural valve degeneration over the follow-up period for FS patients; one patient with a Mitroflow Bioprosthesis was reoperated eight years later due to valve degeneration. Major adverse events, defined by a composite endpoint of death, reoperation, valve infection, and stroke were analyzed, demonstrating no significant difference over the observed time period with a log rank *P*=0.2 (Figure 3).

## DISCUSSION

The high-risk profile of patients with acute infective endocarditis of the aortic valve and root presents a challenging scenario^[1,2]^. Improved antibiotic treatment and modified surgical repair have reduced the relevance of avoiding foreign material, as propagated in the past. Homograft use in the current era is prone to cumbersome implementation: availability remains difficult, the technical challenge of the full-root procedure is complex for patients already presenting with high risk, and long-term durability of the allograft is limited due to accelerating calcification^[10-13]^.

Kim et al.^[14]^ investigated the performance of homografts pitted against mechanical and tissue valves evaluating short- and long-term outcomes. This failed to demonstrate a superiority of the homograft with regard to resistance to reinfection, early death, or overall death. Jassar et al. showed no significant difference when analyzing 134 patients with infective endocarditis treated with mechanical composite graft (32.1%), non-homograft biological valve conduit (41.0%), and homograft (26.9%) regarding re-admission, reinfection, and reoperation, presenting an in-hospital mortality of 18.6% *vs*. 21.8% *vs*. 25% (mechanical *vs*. tissue *vs*. homograft) and comparable survival in five years^[15]^.

Toyoda et al.^[8]^ investigated recurrent infections in patients undergoing mechanical or tissue valve replacement in the setting of endocarditis, failing to demonstrate any significant difference, with similar survival and freedom from endocarditis recurrence (9.4% *vs*. 10.0%, adjusted Cox *P*=0.81 after aortic valve replacement in 12 years).

Stentless bioroots have proven to be a viable alternative, the design facilitating reconstructive procedures at annular level and hemodynamic superiority by omission of the obstructive elements^[16]^. A review by Perrotta et al.^[4,17]^ show stentless valves to have a low reinfection rate of 3.7% to 8.6%, comparable to homografts. Siniawski et al.^[6]^ compared patients receiving Shelhigh prostheses to homografts, showing good results for postoperative gradients and echocardiographic variables. Silaschi et al.^[5]^ demonstrated favorable outcomes of stentless valves when investigated against mechanical valves and stented bioprostheses with no early reinfection < 90 days for bioroot replacement *vs*. 4.4% for mechanical valves and 7.1% for stented tissue valves. Patients with complicated infective endocarditis had a superior long-term survival when treated with stentless valves, possibly by radical debridement leading to greater chance of infect-free tissue when performing root replacement surgery.

Our data, with large volume use of stentless valves, encouraged our approach to treatment of root endocarditis with the xenograft bioroot. The integrity of the functional unit of the aortic root contributes to the excellent long-term performance of the Freestyle valve, and the full-root technique simplifies oversizing and achieves an excellent haemodynamic profile left ventricular remodeling^[16]^. The use of the Freestyle valve in the setting of infective endocarditis was described in smaller case series; Heinz et al.^[18]^ described their experience in 32 patients with a 30-day mortality of 19.4% and a freedom from death, reoperation, and recurrence of infection as a composite endpoint for 56.3% after five years and 53.1% at 10 years^[19]^. Schneider et al.^[20]^ likewise demonstrated a series of 54 patients with excellent results, an early mortality of 11%, and late mortality of 14%. Miceli et al.^[21]^ investigated a small series of 18 patients treated with the stentless Freestyle bioprosthesis with an in-hospital mortality of 11% and freedom from reoperation of 87.5% after two years. These numbers compare well to the data presented here, an in-hospital mortality of 16.7% and a five-year survival of 63%, with one patient presenting with recurrent reinfection 38 months after the initial surgery. These numbers fare well in comparison to the Tissue valve group, with a five-year survival of 47% and likewise one patient reoperated with a full-root replacement for recurrent re-valve surgery for degeneration of the stented bioprosthesis. This data is supported by a previous report from our institution, comparing the FS cohort with a propensity matched group of all surgically treated endocarditis patients^[23]^.

Prosthetic valve infections present as an extremely high-risk cohort. Excision can often lead to significant damage of the annulus requiring complex reconstructive procedures. These patients represent the majority of patients treated with full-root stentless valves. Edlin et al.^[22]^ demonstrated 60% of patients with PVE with an overall mortality of 16% and 53% five-year survival. Schneider et al. likewise demonstrated 54% of the patients treated with the Freestyle full root with PVE^[20]^. Miceli et al.^[21]^ showed 78% of their patients with PVE treated with the Medtronic Freestyle valve with excellent results, with an overall mortality of 11% and a freedom from reoperation of 87.5%. These numbers compare with our data, showing 61.1% of patients with PVE treated with the Medtronic Freestyle valve with an in-hospital mortality of 18.1% of this isolated cohort. The philosophy of root replacement allows suturing of the stentless valve to healthy tissue, excluding the abscess cavity from the bloodstream, possibly with positive influence on long-term freedom from reinfection.

The results of the studies to date suggest patient-specific factors to drive the choice of prosthesis. Operative variables clearly underline the technically more challenging procedure, this however does not negatively influence the early postoperative results in a significant manner. Early mortality was numerically lower in the FS group, however this failed to reach statistical significance.

Long-term results demonstrate a low reinfection rate and good structural valve integrity in comparison to the other valve types implemented. Selection bias in the treatment of infective endocarditis could be overcome by the propensity matching making the cohorts comparable and allowing us to use risk adjustments to evaluate postoperative outcome.

### Limitations

The study design has its flaws, confounding variables cannot be avoided and selection for valve type is not measurable, dependent on several non-documentable cofactors. The study has inherent limitations due to the retrospective design. Although appropriate statistical methods for risk adjustment were implemented, confounding by unmeasured covariates may have affected the results. This study may be underpowered for testing the study hypotheses due to the small number of patients.

## CONCLUSION

Treatment of patients with infective endocarditis of the aortic root remain a formidable task for patient and surgeon with a high mortality rate reflecting the high-risk profile. The use of stentless xenografts in a full-root fashion allow for easier reconstruction at annular level excluding the infective processes out of bloodstream, with a low recurrent reinfection rate over the entire follow-up period. The full-root valve design is beneficial for long-term durability and lack of obstructive elements allow varying therapeutic approaches for treatment of future valve degeneration.

**Table:** 

Authors' roles & responsibilities	
JE	Substantial contributions to the conception of the work; drafting the work; agreement to be accountable for all aspects of the work in ensuring that questions related to the accuracy or integrity of any part of the work are appropriately investigated and resolved; final approval of the version to be published
MS	Substantial contributions to the conception of the work; interpretation of data for the work; drafting the work; final approval of the version to be published
PH	Substantial contributions to the conception of the work; interpretation of data for the work; revising it critically for important intellectual content; final approval of the version to be published
AM	Substantial contributions to the conception of the work; interpretation of data for the work; revising it critically for important intellectual content; final approval of the version to be published
KZ	Substantial contributions to the conception of the work; interpretation of data for the work; drafting the work; final approval of the version to be published
RM	Substantial contributions to the conception of the work; interpretation of data for the work; drafting the work; final approval of the version to be published
RPT	Substantial contributions to the conception of the work; interpretation of data for the work; revising it critically for important intellectual content; final approval of the version to be published
AW	Agreement to be accountable for all aspects of the work in ensuring that questions related to the accuracy or integrity of any part of the work are appropriately investigated and resolved; final approval of the version to be published
OED	Agreement to be accountable for all aspects of the work in ensuring that questions related to the accuracy or integrity of any part of the work are appropriately investigated and resolved; final approval of the version to be published
